# Differences in morbidity and mortality between unilateral adrenalectomy for adrenal Cushing’s syndrome and bilateral adrenalectomy for therapy refractory extra-adrenal Cushing’s syndrome

**DOI:** 10.1007/s00423-022-02568-8

**Published:** 2022-05-28

**Authors:** Joachim Reibetanz, Matthias Kelm, Konstantin L. Uttinger, Miriam Reuter, Nicolas Schlegel, Mohamed Hankir, Verena Wiegering, Christoph-Thomas Germer, Martin Fassnacht, Johan Friso Lock, Armin Wiegering

**Affiliations:** 1 Department of General, Visceral, Transplant, Vascular and Pediatric Surgery at Würzburg University Hospital, Würzburg, Germany; 2 Department of Visceral, Transplant, Thoracic and Vascular Surgery at Leipzig University Hospital, Leipzig, Germany; 3 Division of Endocrinology and Diabetes, Department of Medicine I, University Hospital, University of Würzburg, 97080 Würzburg, Germany; 4 Department of Pediatric Hematology, Oncology and Stem Cell Transplantation, University Children’s Hospital, University of Wuerzburg, Josef-Schneiderstr. 2, 97080 Würzburg, Germany; 5 Comprehensive Cancer Center Mainfranken, University of Würzburg Medical Centre, Würzburg, Germany; 6 Department of Biochemistry and Molecular Biology, University of Würzburg, Würzburg, Germany; 7 Department of General, Visceral, Transplant, Vascular and Paediatric Surgery, Medical Centre, Julius Maximilians University of Würzburg, Oberduerrbacher Strasse 6, 97080 Würzburg, Germany

**Keywords:** Cushing, Adrenal surgery, MTL30, Complication

## Abstract

**Purpose:**

In selected cases of severe Cushing’s syndrome due to uncontrolled ACTH secretion, bilateral adrenalectomy appears unavoidable. Compared with unilateral adrenalectomy (for adrenal Cushing’s syndrome), bilateral adrenalectomy has a perceived higher perioperative morbidity. The aim of the current study was to compare both interventions in endogenous Cushing’s syndrome regarding postoperative outcomes.

**Methods:**

We report a single-center, retrospective cohort study comparing patients with hypercortisolism undergoing bilateral vs. unilateral adrenalectomy during 2008–2021. Patients with adrenal Cushing’s syndrome due to adenoma were compared with patients with ACTH-dependent Cushing’s syndrome (Cushing’s disease and ectopic ACTH production) focusing on postoperative morbidity and mortality as well as long-term survival.

**Results:**

Of 83 patients with adrenalectomy for hypercortisolism (65.1% female, median age 53 years), the indication for adrenalectomy was due to adrenal Cushing’s syndrome in 60 patients (72.2%; 59 unilateral and one bilateral), and due to hypercortisolism caused by Cushing’s disease (*n* = 16) or non-pituitary uncontrolled ACTH secretion of unknown origin (*n* = 7) (27.7% of all adrenalectomies). Compared with unilateral adrenalectomy (*n* = 59), patients with bilateral adrenalectomy (*n* = 24) had a higher rate of severe complications (0% vs. 33%; *p* < 0.001) and delayed recovery (median: 10.2% vs. 79.2%; *p* < 0.001). Using the MTL30 marker, patients with bilateral adrenalectomy fared worse than patients after unilateral surgery (MTL30 positive: 7.2% vs. 25.0% *p* < 0.001). Postoperative mortality was increased in patients with bilateral adrenalectomy (0% vs. 8.3%; *p* = 0.081).

**Conclusion:**

While unilateral adrenalectomy for adrenal Cushing’s syndrome represents a safe and definitive therapeutic option, bilateral adrenalectomy to control ACTH-dependent extra-adrenal Cushing’s syndrome or Cushing’s disease is a more complicated intervention with a mortality of nearly 10%.

## Introduction

Endogenous Cushing’s syndrome (CS) encompasses all conditions of pathologically increased and biologically effective cortisol levels that are not caused by an exogenous administration of glucocorticoids [1]. Long-term CS is associated with various complications arising from severe hypercortisolism including an increased risk of thromboembolism, stroke, insulin resistance, and myocardial infarction, which together contribute to a twofold increased risk of mortality compared to unaffected populations [2]. Endogenous CS is categorized into ACTH-independent and ACTH-dependent forms. ACTH-independent CS, which accounts for 15–20% of all endogenous CS, arises from a cortisol-producing adrenal adenoma [3]. In these cases, unilateral adrenalectomy of the cortisol producing adrenal gland is the treatment of choice with an excellent prognosis [4]. In contrast, for ACTH-dependent CS, a distinction is made between Cushing’s disease (CD) and ectopic CS. In the more common CD, the cortisol excess results from a usually benign ACTH secreting tumor of the pituitary gland [5]. The treatment of choice for the vast majority of patients is trans-sphenoidale pituitary surgery [6]. The second form of ACTH-dependent CS is ectopic CS, caused by non-pituitary neuroendocrine tumors (e.g., small-cell lung carcinomas or lung carcinoids) [7]. In these cases, the most effective treatment is removal of the ACTH-secreting tumor [8]. However, in some cases of ectopic CS, the source of ACTH production remains occult and a bilateral adrenalectomy becomes necessary for effective treatment [9]. In both forms of ACTH-dependent CS, a small subset of patients cannot be cured by surgery of the primary ACTH-producing tumor nor can their hypercortisolism be (long-term) controlled by available drugs. These patients are at higher risk to die on sequel of highly elevated cortisol levels (e.g., infections or thromboembolic disease). Thus, bilateral adrenalectomy is frequently the only therapeutic option for these patients to avoid sequelae.

Since unilateral and bilateral adrenalectomies are considered to have different range of perioperative complications, the aim of the current study was to compare both interventions for endogenous CS regarding short- and long-term postoperative outcomes.

## Methods

We performed a single-center, retrospective cohort study in a 1,500 bed tertiary hospital in Germany.

### Study design

All patients ≥ 18 years undergoing elective adrenalectomy for hypercortisolism since 2008 were included. According to international guidelines, hypercortisolism was defined as pathological results in at least two of the following tests: 1 mg dexamethasone suppression test, bed-time-cortisol, 24 h-urine-cortisol levels, and clinical signs of CS. All relevant data were retrieved from the hospital information system and transferred in a pseudonymous database with multiple variables containing baseline patient characteristics, surgical therapy, postoperative outcomes, and long-term survival. Postoperative complications were graded according to the Clavien-Dindo system. Clavien-Dindo grades I–II complications were considered non-severe, whereas Clavien-Dindo grades IIIa–V complications were considered severe [10, 11]. The primary endpoint was the comprehensive complication index (CCI) [12]. Secondary endpoints were postoperative as well as long-term survival, and two combined clinical endpoints of postoperative outcomes: first, we used the MTL30, a validated marker in colorectal surgery, combining mortality, transfer to another acute care hospital, and length-of-stay (LOS) > 30 days [13, 14]. Secondly, we defined delayed postoperative recovery as a comprehensive complication index (CCI) [12] > 20 points or a prolonged LOS (> 75% percentile, 11 days). Patient descriptions were compared for adrenal CS or extra-adrenal CS including CD. Only a single patient with adrenal CD received bilateral adrenalectomy due to bilateral hyperplasia. In contrast, all patients with extra-adrenal CS or CD received bilateral adrenalectomy. Therefore, the analysis of outcome compared unilateral vs. bilateral adrenalectomies.

### Surgical procedures

Minimally invasive transabdominal adrenalectomy was performed in lateral position of the patient on a vacuum mattress. Left-sided adrenalectomy was performed using a three-trocar technique, the right-sided adrenalectomy using a four-trocar technique, since an additional trocar is required here to expose the right lobe of the liver. The camera trocar was placed transrectally at the level of the umbilicus, a further 5 mm trocar just above the anterior superior iliac spine and a further 12 mm trocar subcostally. (For right-sided adrenalectomy, another 10 mm trocar for the liver retractor was placed in the epigastric region). Access for open adrenalectomy (*n* = 6) was via a transverse subcostal incision.

### Perioperative corticoid supplementation

All patients received a bolus of 100 mg hydrocortisone with induction of anesthesia, followed by continuous perfusion of 100 mg/24 h on the 1st postoperative day. Patients with severe CS received 200 mg/24 h hydrocortisone. After the start of oral intake, the patient switched to oral hydrocortisone at a dosage of 30–20-10 mg. In general, the duration of corticoid supplementation depended on the severity of preoperative CD and lasted between 6 and 18 months. During this time, the dose was reduced in steps of 10 mg (later 5 mg). At a daily dose of 20 mg, an ACTH stimulation test was carried out.

### Statistical analysis

All statistical analysis was performed using IBM SPSS Statistics, version 26 (International Business Machines Corporation, Armonk, NY). Descriptive data were reported as medians with range unless otherwise noted. Statistical tests were applied according to the data distribution using Mann–Whitney *U* test or analysis of variance for independent samples, and chi-square test or Fisher’s exact test for categorical variables. Survival analysis was performed using Kaplan–Meier analysis. Multivariate analysis of combined endpoint delayed postoperative recovery was performed by logistic regression and of survival by Cox regression analysis. Two-sided *p* values < 0.05 were considered statistically significant.

## Results

From a local database of adrenalectomy, we identified in total 83 patients with adrenalectomy for hypercortisolism during 2008–2021. Sixty-five percent were female, the median age was 53 (range: 23–76) years, and median body mass index was 28.0 (range: 18.8–50.5) kg/m^2^. The prevalence of common comorbidities including hypertension, type II diabetes, chronic kidney failure, hypokalemia, and osteoporosis are presented in Table 1. In 73 patients (88%), a preoperative dexamethasone suppression test was performed with pathological results in 63 patients (86.3%).

**Table 1:** Patients’ and operative characteristics

	Total (*n* = 83)	Adrenal (*n* = 60)	Extra-adrenal (*n* = 23^4^)	*p*-value
Age, years^1^	53 (23–76)	52.5 (23–76)	59 (27–76)	0.294
Sex, male	29 (34.9)	16 (26.7)	13 (56.5)	0.011
BMI^1^	28.0 (18.8–50.5)	28.2 (19.9–50.5)	26.2 (18.8–46.1)	0.663
ASA classification				
II	48 (57.2)	40 (66.7)	8 (36.4)	0.055
II	28 (33.7)	17 (28.3)	11 (50.0)	
IV	5 (6)	2 (3.3)	3 (13.6)	
Size of adrenal gland/tumor, cm^1^	3.5 (1.3–11.0)	3.5 (1.8–11.0)	3.0 (2.1–5.5)^5^	0.97
ACTH-secreting malignant tumors	7 (8.4%)	0	7 (30.4)	< 0.001
History of adrenal disease, months^1^	7.5 (1–146)	7.8 (1–146)	4 (1–88)	0.115
Overt Cushing syndrome^2^	71 (85.5)	48 (80)	23 (100)	0.020
Baseline serum cortisol, µg/dl^1^	17.6 (5.4–273)	15.2 (5.4–273)	26.3 (11.2–144)	< 0.001
Baseline ACTH level, ng/l^3^	6.3 (5.0–460)	5.2 (5.0–7.5)	140 (41–182)	< 0.001
Diabetes mellitus	28 (33.7)	15 (25)	13 (56.5)	0.007
HbA1c, %^1^	5.9 (4.5–10.5)	5.8 (4.5–10.2)	6.3 (5.0–10.5)	0.016
Hypertension	61 (73.5)	44 (73.3)	17 (73.9)	0.96
CKD	8 (9.6)	3 (5)	5 (21.7)	0.034
Hypokalemia	13 (15.7)	3 (5)	10 (43.5)	< 0.001
Osteoporosis	15 (18.1)	9 (15)	6 (26.1)	0.34
Preoperative LOS, days^1^	1 (0–62)	1 (0–4)	11 (0–62)	< 0.001
Laparoscopy	77 (92.8)	56 (93.3)	21 (91.3)	0.75
Bilateral adrenalectomy	24 (28.9)	1 (1.7)	23 (100)	< 0.001
Duration of surgery, minutes^1^	83 (29–344)	64 (29–235)	162 (83–344)	< 0.001
Estimated blood loss, ml^1^	< 50 (0–800)	< 50 (0–800)	< 50 (0–100)	1

^1^Median with range; ^2^clinical overt symptoms of Cushing’s syndrome; ^3^median with interquartile range; ^4^*n* = 16 patients with Cushing’s disease, *n* = 7 with ectopic ATCH producing malignant tumors (occult ACTH syndrome); ^5^in patients with Cushing’s disease and occult ACTH syndrome who do not show an actual adrenal tumor, the longest diameter of the adrenal gland was measured according to imaging

Abbreviations: *BMI*, body mass index in kg/m.^2^; *ASA* American Society of Anesthesiologists; *ACTH*, adrenocorticotropic hormone; *CKD*, chronic kidney disease; *LOS*, length-of-stay

The indication for adrenalectomy was adrenal CS due to an adrenal adenoma in 60 patients (72.2%; 59 unilateral and one bilateral hyperplasia). In 23 patients (27.7%), a bilateral adrenalectomy was indicated: in 16 patients for CD and in 7 patients for ectopic ATCH producing malignant tumors (occult ACTH syndrome).

**Fig. 1:**
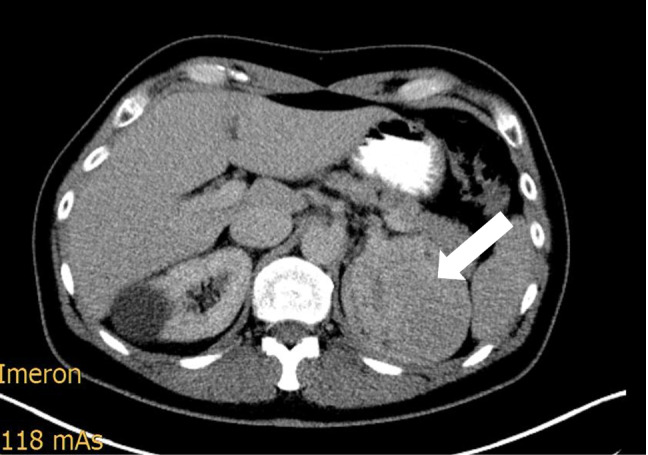
Exemplary CT scan of a female patient with a 9.5 cm left cortisol-producing adrenal adenoma

Compared with adrenal CS, patients with extra-adrenal indication for bilateral adrenalectomy were more commonly male, had higher cortisol and ACTH levels, presented with more severe comorbidities, and had higher rates of malignancy (Table 1). No differences concerning the tumor size were observed between the two groups (Fig. 1 displays an example of a large adrenal tumor in CS).

The overall operation times were significantly longer for bilateral adrenalectomy, whereas no differences were found for percentage of open procedures or estimated blood loss. In total, open procedures were performed in 6 patients (1 conversion because of intraoperative bleeding from a segmental branch of the renal artery, 3 due to tumor size larger then 8–10 cm (Fig. 1), and 2 due to prior abdominal surgery).

Parameters for postoperative outcomes are presented in Table 2. Patients with bilateral adrenalectomy had a significantly prolonged median LOS at the intensive care unit and total LOS (1 vs. 3 days; *p* < 0.001, and 6 vs. 14 days; *p* < 0.001). For bilateral adrenalectomies, a higher rate of complications as well as more severe complications (Clavien-Dindo, CDC ≥ III: 0 vs.33.3%; *p* < 0.001) were documented. Accordingly, the comprehensive complication index (CCI) was significantly higher in these patients, compared to patients after unilateral adrenalectomy (20.9 (range: 0–30.8) versus 0 (range: 0–0), *p* < 0.001; Table 2). While the in-house mortality rate for patients with adrenal CS was 0%, two of the 23 (8.3%) suffered from postoperative death in the group of bilateral adrenalectomy (*p* = 0.081). Both patients received uneventful laparoscopic procedures. One patient developed fatal mesenteric ischemia (non-occlusive disease as a consequence of CS cardiomyopathy) on postoperative day 3, while the second patient suffered from severe postoperative hemorrhage (diffuse bilateral bleeding with a large intraabdominal hematoma) requiring laparotomy followed by multiple organ dysfunction syndrome and death 58 days after surgery.

**Table 2:** Postoperative outcomes

	Total (*n* = 83)	Unilateral (*n* = 59)	Bilateral (*n* = 24)	*p*-value
LOIS, days^1^	1 (0–56)	1 (1–8)	3 (1–56)	< 0.001
LOS, days^1^	7 (3–58)	6 (3–27)	14 (3–58)	< 0.001
SSI	7 (8.4)	2 (3.4)	5 (20.8)	0.008
Other infections	4 (4.8)	1 (1.7)	3 (12.5)	0.071
Addison crisis	4 (4.8)	1 (1.7)	3 (12.5)	0.071
CDC ≥ grade IIIa	8 (9.6)	0	8 (33.3)	< 0.001
CCI^2^	0 (0–8.7)	0 (0–0)	20.9 (0–30.8)	< 0.001
Perioperative mortality	2 (2.4)	0	2 (8.3)	0.081
MTL30	6 (7.2)	0	6 (25.0)	< 0.001
Delayed postoperative recovery^3^	25 (30.1)	6 (10.2)	19 (79.2)	< 0.001

^1^Median with range; ^2^median with quartiles; ^3^compisite endpoint of CCI > 20 pts. OR LOS > 11 days (75% percentile)

Abbreviations: *LOIS*, length of intensive care stay; *LOS*, length of postoperative stay; *SSI*, surgical site infections; *CDC*, Clavien-Dindo classification of surgical complications; *CCI*, comprehensive complication index; *MTL30*; mortality-transfer-LOS > 30

As no postoperative mortality as well as major complications occurred in the adrenal Cushing group, we analyzed two combined endpoints as secondary outcome: MTL30 and “delayed recovery.” In the bilateral adrenalectomy group, a significant higher percentage of patients were MTL30 positive (7.2% vs. 25.0%; *p* < 0.001). Secondly, in the endpoint marker termed “delayed recovery,” 10% of patients undergoing unilateral adrenalectomy due to adrenal hypercortisolism showed delayed recovery, whereas nearly 80% of patients who were operated on for extra-adrenal CS showed delayed recovery according to the abovementioned definition (*p* < 0.001, Table 2).

**Fig. 2:**
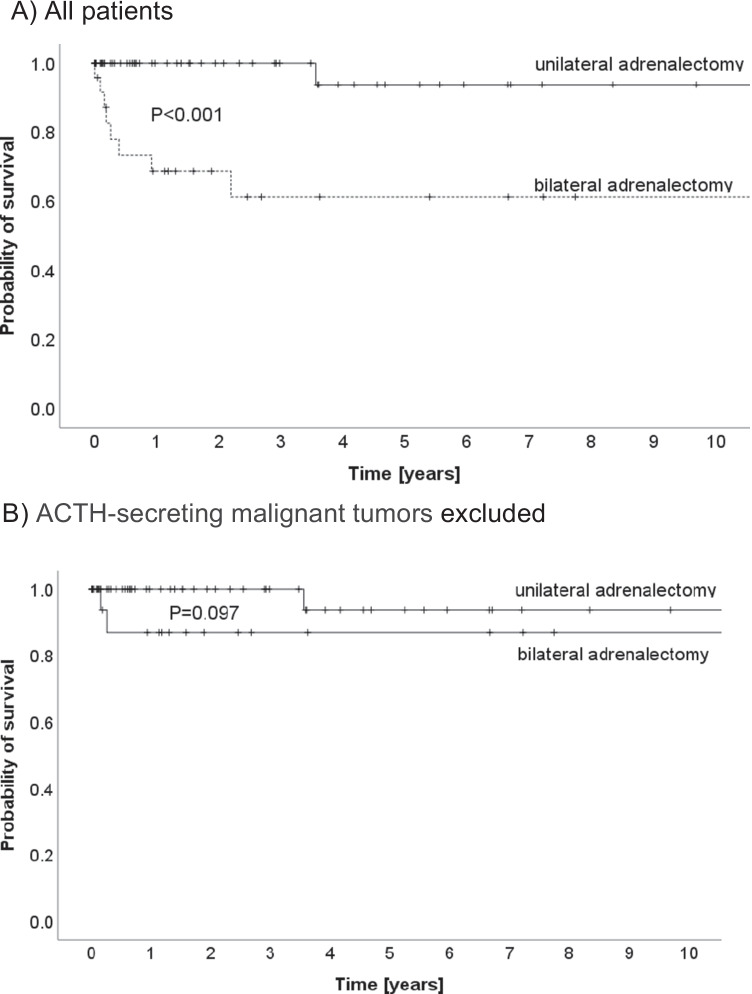
10-year survival analysis. **A** All patients. **B** ACTH-secreting malignant tumors excluded. The two patients in the unilateral adrenalectomy group who died almost 4 years after initial adrenalectomy died from a metastatic cancer not related to adrenal disease

Kaplan–Meier estimated median survival was significantly longer after unilateral adrenalectomy as compared to bilateral adrenalectomy (12.5 years (95% CI 11.4–13.6 years) versus 7.4 years (95% CI 5.0–9.8); *p* < 0.001; Fig. 2A). When patients with ACTH-secreting malignant tumors were excluded (*n* = 7) from analysis, no significant difference in survival was observed (12.5 years vs. 10.2 years; *p* = 0.097; Fig. 2B). The two patients in the unilateral adrenalectomy group who died almost 4 years after initial adrenalectomy died from metastatic cancer not related to adrenal disease.

Multivariable analysis indicated laparotomy (odds ratio 15.1; *p* = 0.019) and bilateral adrenalectomy as predictors of predictors of delayed postoperative recovery (odds ratio 56; *p* < 0.001). In contrast, no significant impact of age, severity, or preoperative duration of CD on postoperative recovery could be observed.

## Discussion

In this retrospective analysis, we could clearly show that adrenalectomy for adrenal CS due to adrenal adenoma is a safe and definitive therapeutic option with zero perioperative mortality and excellent long-term survival rates. However, bilateral adrenalectomy to control for ACTH-dependent extra-adrenal CS had disproportionately higher complication rates and a high mortality rate of nearly 10%.

This difference in outcome might be explained by the following: while adrenalectomy for adrenal CS is a well-standardized procedure and the first-line therapy, bilateral adrenalectomy for extra-adrenal CS is mostly a fail-safe palliative intervention that reduces the serious consequences induced by hypercortisolism in an otherwise non-curable disease. Comparable with uncontrolled hypercortisolism, patients with extra-adrenal CS presented with higher ACTH and cortisol levels, and higher percentage of malignancy, and were more likely to suffer from secondary problems of the hypercortisolism like type II diabetes or hypokalemia. These results are in line with previous studies showing that approximately 80% and 60% of patients with ectopic Cushing syndrome have hypertension or hypokalemia, respectively [15]. Interestingly, we found only a slightly longer time interval from the first diagnosis of hypercortisolism to surgical therapy in adrenal and extra-adrenal CS (7.8 vs. 4 months). This is in contrast to previous studies in which the time interval from the first diagnosis to adrenalectomy was 54 months for bilateral adrenalectomy for CD versus 4 months in patients with ectopic ACTH-producing tumors [16]. Our results in this context are unexpected since bilateral adrenalectomy in ectopic CS is usually the eventual treatment after extensive diagnostics and conservative therapies. However, we report results of a highly specialized endocrine unit where the diagnostic pathway is presumably shorter compared to a less specialized outpatient center.

In large population-based studies, surgical intervention of CS and hypercortisolism alone fails to completely alleviate the negative consequences of the disease, and mortality remains increased in the long-term even in patients thought to be cured from CS [2]. This suggests that cardiovascular risk is increased even after successful surgery for CS [17]. Additionally, our study indicates that there might be differences in the long-term prognosis between adrenal and extra-adrenal CS. While in our study patients with adrenal Cushing adenoma had an excellent long-term prognosis, the survival of patients with bilateral adrenalectomy for extra-adrenal CS was worse. However, the latter observation might be truly explained by the higher percentage of patients suffering from malignant disease within the group of bilateral adrenalectomies (30% versus 0%). When adjusting for this factor, the difference in long-term survival disappeared arguing that bilateral adrenalectomy has a worse prognosis. Thus, postoperative long-term prognosis of CD with bilateral adrenalectomy was as good as for unilateral adrenalectomy for CS. This observation is supported by the literature [18]. Bilateral adrenalectomy in patients with otherwise uncontrolled hypercortisolism in most cases leads to very good and immediate control of hypercortisolism. The Cushing’s associated signs and symptoms are corrected with a favorable impact on comorbidity [18]. Accordingly, a systematic review on the outcome of bilateral adrenalectomy in CS reports an excellent degree of clinical disease remission: muscular weakness improves in 93%, phenotypic stigmata in 77%, arterial hypertension in 80%, and diabetes mellitus in 75% of patients. In this study, too, mortality after bilateral adrenalectomy was mainly reported in the early postoperative period (up to 12 months), mostly as a result of stroke, myocardial infarction, or septicemia [19]. These and our data show that the perioperative period after bilateral adrenalectomy can be critical, but that patients benefit significantly more from the long-term control of hypercortisolism than they would be at risk of a possible adrenal crisis after bilateral adrenalectomy (on average 28% after 42 months, [19].

However, all patients with ACTH-secreting malignant tumors deceased within 2 years after operation. This must be taken into account in the preoperative decision making and indication for salvage bilateral adrenalectomy.

Compared to previous series on laparoscopic adrenalectomy, the inpatient stays in our study (6 days in unilateral adrenalectomy, 14 days in bilateral adrenalectomy) appear to be longer. This is, on the one hand, due to the circumstances of the German healthcare system. On the other hand, and mainly, patients with hypercortisolism are particularly vulnerable perioperatively and are monitored in our institution until any surgical complications have been ruled out. Especially in the case of patients after bilateral adrenalectomy, it has been established in our clinic that patients are transferred to the endocrinology department on the third postoperative day. This is where the further care, hormone adjustment, and training of the patients with regard to the lost adrenal function take place.

Our study has several limitations. The retrospective design is prone to some bias and therefore, conclusions must be drawn carefully. Furthermore, due to the fact that adrenalectomy (for CS) is an overall rare operation, our study is probably underpowered to show slight differences between the two cohorts [20]. Nevertheless, despite the small size of our study, we were able to demonstrate a significantly higher rate of postoperative complications in the extra-adrenal Cushing group, suggesting that there is an obvious difference in perioperative outcome of unilateral and bilateral adrenalectomies, even in a specialized unit. In this aspect, it must be considered that our center has ample expertise for diseases of the adrenal gland where more than 60 adrenal resections are performed per year. In line with other operative procedures, it was shown that the postoperative mortality for adrenalectomy correlates with the annual hospital volume [21–26], arguing that especially adrenalectomy for extra-adrenal CS should be performed in specialized centers.

In conclusion, we could demonstrate that if indicated, bilateral adrenalectomy is a feasible option for otherwise uncontrolled hypercortisolism. However, the disproportionately high morbidity and mortality of this intervention must be considered.

## Data Availability

Not applicable.
