# Correction to: Differences in morbidity and mortality between unilateral adrenalectomy for adrenal Cushing’s syndrome and bilateral adrenalectomy for therapy refractory extra-adrenal Cushing’s syndrome

**DOI:** 10.1007/s00423-022-02601-w

**Published:** 2022-07-13

**Authors:** Joachim Reibetanz, Matthias Kelm, Konstantin L. Uttinger, Miriam Reuter, Nicolas Schlegel, Mohamed Hankir, Verena Wiegering, Christoph-Thomas Germer, Martin Fassnacht, Joha Friso Lock, Armin Wiegering

**Affiliations:** 1 Department of General, Visceral, Transplant, Vascular and Pediatric Surgery at Würzburg University Hospital, Würzburg, Germany; 2 Department of Visceral, Transplant, Thoracic and Vascular Surgery at Leipzig University Hospital, Leipzig, Germany; 3 Division of Endocrinology and Diabetes, Department of Medicine I, University Hospital, University of Würzburg, 97080 Würzburg, Germany; 4 Department of Pediatric Hematology, Oncology and Stem Cell Transplantation, University Children’s Hospital, University of Wuerzburg, Josef-Schneiderstr. 2, 97080 Würzburg, Germany; 5 Comprehensive Cancer Center Mainfranken, University of Würzburg Medical Centre, Würzburg, Germany; 6 Department of Biochemistry and Molecular Biology, University of Würzburg, Würzburg, Germany; 7 Department of General, Visceral, Transplant, Vascular and Paediatric Surgery, Medical Centre, Julius Maximilians University of Würzburg, Oberduerrbacher Strasse 6, 97080 Würzburg, Germany

**Correction to: Langenbeck's Archives of Surgery**

10.1007/s00423-022-02568-8

The original version of this article unfortunately had incomplete details on references 24–26. The corrected items are shown in the following References section.

The original article has been corrected.
